# Transgenerational plasticity in a zooplankton in response to elevated temperature and parasitism

**DOI:** 10.1002/ece3.9767

**Published:** 2023-02-03

**Authors:** Syuan‐Jyun Sun, Marcin K. Dziuba, Riley N. Jaye, Meghan A. Duffy

**Affiliations:** ^1^ Department of Ecology and Evolutionary Biology University of Michigan Ann Arbor Michigan USA; ^2^ International Degree Program in Climate Change and Sustainable Development National Taiwan University Taipei Taiwan

**Keywords:** adaptation, *Daphnia*, environmental stressors, host–parasite interactions, *Metschnikowia*, transgenerational plasticity

## Abstract

Organisms are increasingly facing multiple stressors, which can simultaneously interact to cause unpredictable impacts compared with a single stressor alone. Recent evidence suggests that phenotypic plasticity can allow for rapid responses to altered environments, including biotic and abiotic stressors, both within a generation and across generations (transgenerational plasticity). Parents can potentially “prime” their offspring to better cope with similar stressors or, alternatively, might produce offspring that are less fit because of energetic constraints. At present, it remains unclear exactly how biotic and abiotic stressors jointly mediate the responses of transgenerational plasticity and whether this plasticity is adaptive. Here, we test the effects of biotic and abiotic environmental changes on within‐ and transgenerational plasticity using a *Daphnia*–*Metschnikowia* zooplankton‐fungal parasite system. By exposing parents and their offspring consecutively to the single and combined effects of elevated temperature and parasite infection, we showed that transgenerational plasticity induced by temperature and parasite stress influenced host fecundity and lifespan; offsprings of mothers who were exposed to one of the stressors were better able to tolerate elevated temperature, compared with the offspring of mothers who were exposed to neither or both stressors. Yet, the negative effects caused by parasite infection were much stronger, and this greater reduction in host fitness was not mitigated by transgenerational plasticity. We also showed that elevated temperature led to a lower average immune response, and that the relationship between immune response and lifetime fecundity reversed under elevated temperature: the daughters of exposed mothers showed decreased fecundity with increased hemocyte production at ambient temperature but the opposite relationship at elevated temperature. Together, our results highlight the need to address questions at the interface of multiple stressors and transgenerational plasticity and the importance of considering multiple fitness‐associated traits when evaluating the adaptive value of transgenerational plasticity under changing environments.

## Graphical abstract

Different environmental stressors, including biotic and abiotic, can interact and cause unpredictable impacts. Yet, it is unclear when transgenerational effects might help or hinder the fitness of the next generation. Our study shows the evidence of stressor‐induced transgenerational plasticity, but its adaptive significance depends on the identity and combinations of environmental stressors.

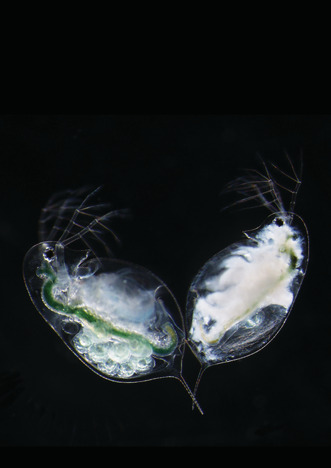

## 1 INTRODUCTION

Understanding how populations and species respond to altered environments is critical in a rapidly changing world (de Laender et al., 2016; García et al., 2018). Adaptation can help organisms cope with environmental changes (Fox et al., 2019) but can require relatively long time scales that may not allow species to keep up with the pace of change (Radchuk et al., 2019; Visser, 2008). Fortunately, phenotypic plasticity can allow organisms to weather the negative impacts of changing environments on shorter time scales (Snell‐Rood et al., 2018), with studies of single stressors showing that phenotypic plasticity can increase fitness in changing environments and even facilitate rapid adaptation (Chevin & Hoffmann, 2017; Levis & Pfennig, 2016; Sun et al., 2020). Phenotypic plasticity can not only influence responses within generations but also across generations (i.e., transgenerational plasticity or maternal effects). Transgenerational plasticity is particularly important for offspring to buffer the adverse impacts of the immediate environment, especially when the environmental cues experienced by previous generations match those of the offspring generation (Mousseau & Fox, 1998). In short, transgenerational plasticity has the potential to allow organisms to cope with the same or different stressors across generations (Garbutt et al., 2014; Meng et al., 2021; Tran et al., 2019).

Environmental stressors, such as temperature increase, land use change, and toxicants, often occur simultaneously and can interact in complex and unpredictable ways (Jackson et al., 2021; Schäfer & Piggott, 2018; Simmons et al., 2021). A growing body of work in multiple‐stressor research has focused on understanding and predicting interactions between different stressors, which can cause antagonistic or synergistic effects compared with an individual stressor (Orr et al., 2020). Moreover, these responses can occur across generations, with the potential for parents to “prime” their offspring to better handle stressful environments (Tran et al., 2019). While it is clear that transgenerational plasticity can impact offspring fitness in the face of multiple stressors, to date studies have focused primarily on abiotic stressors. While understanding abiotic stressors—perhaps most notably the temperature regimes that are increasingly common as a result of anthropogenic climate change—is extremely important, it is also important to recognize that biotic factors (e.g., parasites and predators) routinely shift alongside temperature and other abiotic factors. Thus, in order to fully understand how global change will impact organisms, we must study the joint impact of abiotic and biotic stressors.

A long‐standing idea is that climate warming may exacerbate the negative effects of parasites, partly because elevated temperatures increase the fitness of the parasites and/or weaken host defenses (Harvell et al., 2002). However, warming effects on parasites are likely nonlinear, such that extremely warm temperatures can cause reduced parasite performance (Claar & Wood, 2020; Cohen et al., 2017; Paull & Johnson, 2014). Moreover, studies of multiple stressors show that it can be challenging to predict whether a combination of stressors will increase or decrease the impact of a given stressor (Orr et al., 2020; Piggott et al., 2015). In aquatic species, for example, warming can increase the toxicity of several pesticides (Moe et al., 2013; Noyes et al., 2009) but, in other cases, can decrease pesticide toxicity due to more rapid degradation (op de Beeck et al., 2017). In addition, studies of the joint effects of elevated temperature and parasitism have generally overlooked the possibility that transgenerational effects might alter the impact of these stressors. Host parents who are challenged by parasites can potentially enhance the immune responses of offspring generation when challenged by the same parasites, a type of transgenerational plasticity also known as “transgenerational immune priming” (Paraskevopoulou et al., 2022; Sadd et al., 2005; Tetreau et al., 2019). However, while it is clear that multiple stressors can interact with one another, and that transgenerational plasticity can impact offspring fitness in the face of stressors, most studies of transgenerational plasticity to date have focused on single biotic or abiotic factors (but see Garbutt et al., 2014; Hector et al., 2021; Roth & Landis, 2017), leaving a gap in understanding transgenerational effects in the context of multiple‐stressor research.

Transgenerational plasticity in the face of multiple stressors might increase offspring fitness, especially when the two stressors involve similar physiological mechanisms and when they are predictable. The coordinated physiological responses to environmental stressors can be achieved when one stressor activates signaling pathways for protecting against different stressors, or when different stressors induce independent activation, resulting in overlapping protection (Sinclair et al., 2013). Temperate and polar insects, for example, can better survive the winter at low temperatures and low water availability when these stressors induce similar cellular mechanisms (Sinclair et al., 2013). By contrast, two distinct forms of stressors may hinder the adaptive value of transgenerational plasticity not only because the reduced likelihood that multiple environmental variables match across generations but also because protecting against one stressor might increase vulnerability to another; for example, shifts in temperature in combination with induced pathogen prevalence elevated the energetic costs that are required for acclimation (Roth & Landis, 2017).

In this study, we tested for within‐ and transgenerational effects of abiotic and biotic environmental changes, namely elevated temperature and parasite infection, on host performance using a *Daphnia*–*Metschnikowia* zooplankton‐fungal parasite system. *Daphnia* spp. are particularly ideal for testing transgenerational plasticity in response to stressors because they have a relatively short generation time, and also because they reproduce through cyclical parthenogenesis (Ebert, 2005); this allows us to keep genotype constant across generations, making it possible to attribute any detected differences to environmentally induced host responses. Our focal parasite was the virulent fungus *Metschnikowia bicuspidata*, which reduces host fecundity and lifespan (Clay et al., 2019). Upon host death, *M. bicuspidata* spores are released to the water column where they can be transmitted to healthy *Daphnia* who ingest the fungal spores while foraging for phytoplankton food (Ebert et al., 2000). Transmission of *M. bicuspidata* only occurs horizontally; offspring of infected mothers are not infected.

**FIGURE 1:**
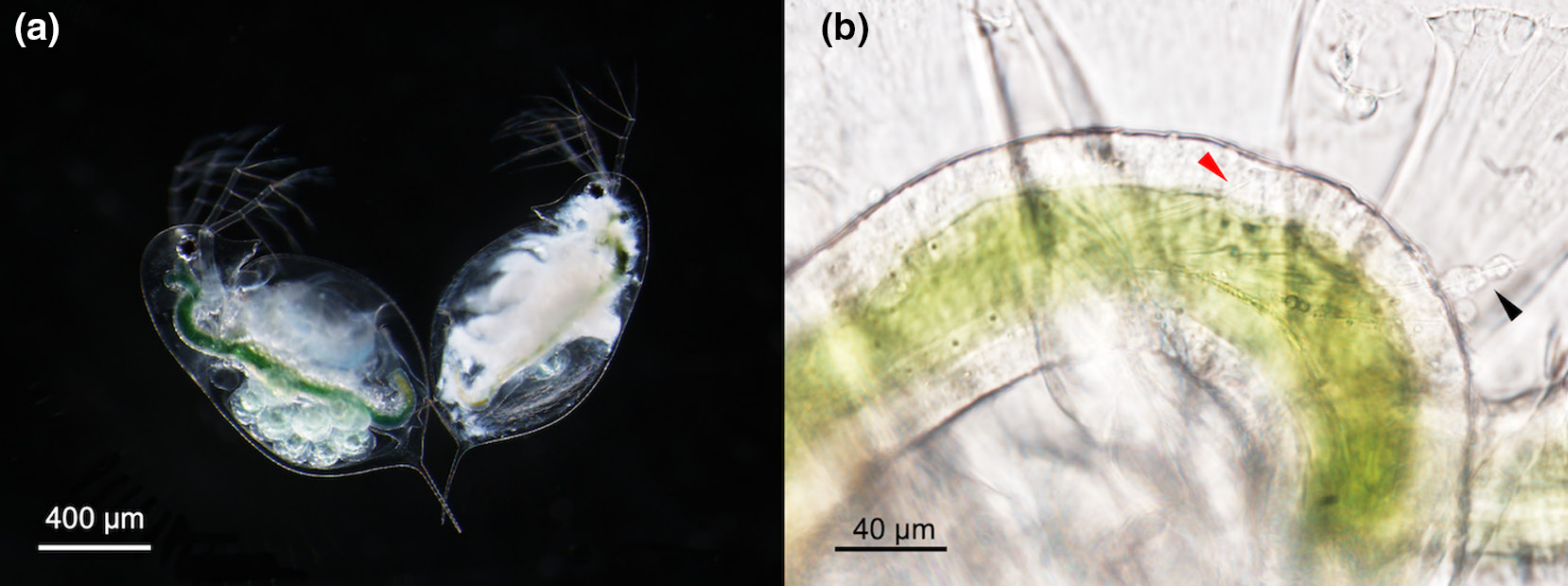
Infection of the water flea *Daphnia dentifera* by the fungal parasite *Metschnikowia bicuspidata*. (a) A comparison of a healthy (left) and a parasite‐infected (right) *D. dentifera*. The uninfected animal holds asexual developing embryos in its brood chamber, while the chamber of the infected animal remains empty. (b) A midgut of *D. dentifera* infected with *Metschnikowia* spores. The red and black arrows indicate an embedded and a fully penetrated hemocoel spore, respectively. *Credit*: Syuan‐Jyun Sun.

Our study focused on the crustacean *Daphnia dentifera* (Figure 1), which is commonly found in stratified lakes in temperate regions in Northern America (Tessier et al., 2002). In these lakes, epidemics typically begin during late summer/early fall (Shocket et al., 2018, 2019). Lakes in this region have increased in temperature by 0.5–1.0°C relative to 1951–1980 (Piccolroaz et al., 2020), with further increases expected, including a 3–25× increased likelihood of severe lake heatwaves with 1.5–3.5°C warming (Woolway et al., 2022). We used a 4°C increase in temperature to simulate a projected warming scenario.

In this study, we examined the single and combined effects of mean temperature elevation and parasite exposure in the parental generation and investigated their offspring's response to the single and combined effects of elevated temperature and parasite infection. This experiment relates to, but differs from, two other recent experiments. In the first (Sun, Dziuba, Jaye, et al., 2022), we focused on how temperature‐modified trait‐mediated infection outcomes in the F0 generation and did not look across generations. In the second related experiment (Sun, Dziuba, Mclntire, et al., 2022), we looked for evidence of transgenerational plasticity in the parasite (rather than in the host, which is the focus of the present study). In the present study, we were interested in host responses and focused on six key host traits. Because fitness is strongly linked with lifespan and reproduction, we measured host lifespan and three components of host reproduction (age at first reproduction, first clutch size, and lifetime fecundity); lifetime fecundity is the most comprehensive measure of host fitness, but the age at first reproduction and first clutch size have a disproportionately large impact on population growth parameters, so we measured those as well. Because we were interested in infection, we also measured two traits related to host immune responses (gut resistance and hemocytes per spore). To study the impacts of changes on the host–parasite interaction, we also measured two traits related to parasite fitness (the probability of terminal infection and spore yield per host); parasite fitness requires successfully infecting a host and causing a terminal infection (which is defined as an infection that produces mature transmission stages; Stewart Merrill et al., 2019) and is correlated with the number of transmission stages (that is, spores) produced at the end of infection. We hypothesized that hosts should produce offspring that are primed to live in similar environments, and thus perform better in their reproduction and/or survival than unprimed offspring (the “environmental matching hypothesis”), with a corresponding decrease in parasite performance. Alternatively, host parents challenged with stressful environments might have less fit offspring, regardless of the type of stressor, due to reduced resources for reproduction (the “stress hypothesis”). Furthermore, we hypothesized that elevated temperature and parasite infection of hosts would have an interactive effect on offspring performance.

## 2 MATERIALS AND METHODS

### 2.1 Experimental setup

**FIGURE 2:**
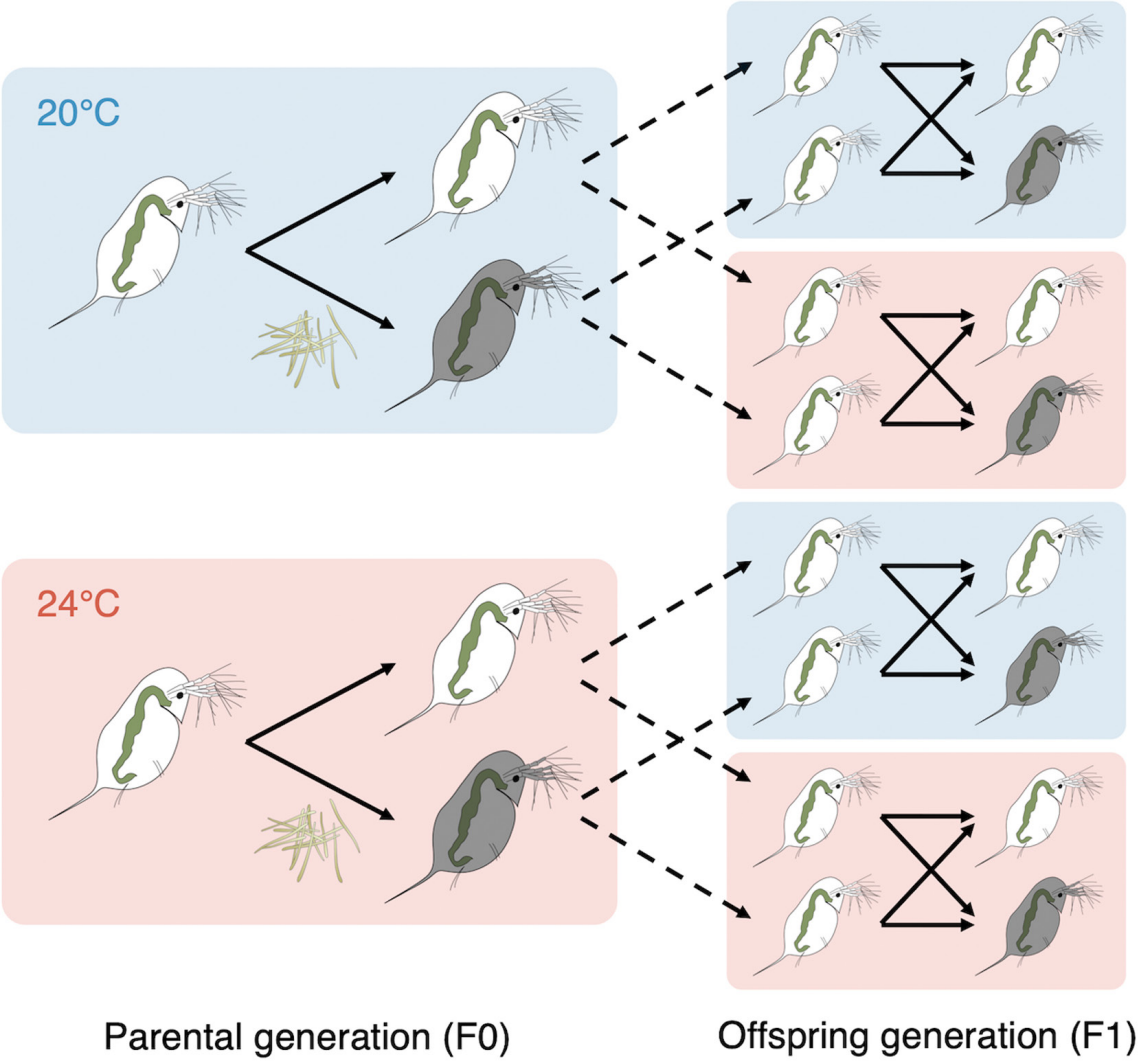
Experimental design used to evaluate whether the single and combined effects of temperature and parasite exposure experienced during parental generations (F0) influenced the performance of offspring (F1) and whether this effect depended on the environment of the offspring. Blue shading indicates ambient temperature (20°C) and red shading indicates elevated temperature (24°C). Solid lines indicate individuals from a given generation being divided between parasite exposure (gray *Daphnia dentifera*) or placebo exposure (white *D. dentifera*). Dashed lines indicate offspring collected from the F0 generation that was used for the F1 generation treatments.

Assessing the adaptive significance of transgenerational plasticity in response to the single or combined effects of environmental stressors requires a fully factorial design manipulating each stressor in both parental and offspring generations (Donelson et al., 2018). This approach allows the fitness components to be fully dissected to evaluate the adaptive value of within‐ and transgenerational effects when parental and offspring environments are matched or mismatched. Therefore, to test for within‐ and transgenerational effects of elevated temperature and/or parasite exposure or infection, we conducted a fully factorial experiment over two generations (Figure 2). This experiment used the “Standard” lab lines of *D. dentifera* and *M. bicuspidata* originally isolated from a lake in Barry County, Michigan. We describe the maintenance of the *D. dentifera* and *M. bicuspidata* used in this study in more detail elsewhere (Sun, Dziuba, Jaye, et al., 2022). Immediately prior to this experiment, *D. dentifera* were maintained in standardized conditions (a 16:8 photoperiod at 22°C) for three generations and fed three times a week with a phytoplankton food (*Ankistrodesmus falcatus*, 20,000 cells/ml). *M. bicuspidata* spores (2 weeks–1 month old) were harvested from *D. dentifera* previously infected by *M. bicuspidata* at an exposure density of 250 spores/ml. Infected *D. dentifera* were stored in a refrigerator before use and were ground up prior to exposure using a cordless pellet pestle (Fisherbrand; Fisher Scientific).

In the parental generation (F0), *Daphnia* were exposed to one of the four treatment combinations that factorially combined elevated temperature (20 and 24°C) and parasite exposure (control/exposed). We collected neonates from the second clutch of the acclimated *D. dentifera* stock populations on the day of birth and placed them either at 20 or 24°C. Each animal was kept individually in a 50 ml beaker filled with 50 ml lake water and fed three times a week (20,000 cells/ml *A. falcatus*). For the parasite exposure treatment, we added *M. bicuspidata* spores at a density of 145 spores/ml to each beaker when juveniles were 6 days and 5 days old at 20 and 24°C, respectively. This degree‐day approach allows for the same accumulated product of time and temperature at degree‐day 120 (Manzi et al., 2020; Vale et al., 2008), thus minimizing potential differences in body size between temperature treatments (as confirmed statistically: *χ* ^2^ = 2.19, df = 1, *p =* .139). We used this approach because we already know that body size can have substantial impacts on host–parasite interactions (Hall et al., 2007) and we wished to isolate the effects of temperature that were not driven by body size. For the unexposed animals, a placebo solution containing the same amount of dead uninfected *D. dentifera* was added to each beaker. The animals were exposed to either the parasite or placebo solution for 24 h, fed 20,000 cells/ml *A. falcatus*, and kept at 16:8 light:dark cycle. All experimental animals were then transferred to new beakers filled with 50 ml spore‐free lake water, fed 20,000 cells/ml *A. falcatus*, and maintained at 16:8 light:dark until the end of the experiment. To test for within‐ and transgenerational plasticity in the offspring generation (F1), we collected neonates from the second and third clutches of F0 adults. We used a split brood design in which four neonates from a single brood were haphazardly selected and one individual assigned to each of the four treatment combinations (two temperature treatments [20 and 24°C] and two parasite exposure treatments [control/exposed]). Because we could not tell whether parents who had been exposed to parasites were successfully infected at the time we collected their offspring, we were unable to discriminate between F0 parents who were infected versus exposed‐but‐uninfected. The experiment was conducted in the same manner in the offspring generation as in the parental generation, and the degree‐day approach once again led to similar body sizes between temperature treatments (*χ* ^2^ = 0.79, df = 1, *p =* .375). In total, there were 16 different treatment combinations (Figure 2), with a total of 248 F1 animals tested (*n* = 68 for 20°C/control, *n* = 56 for 20°C/exposed, *n* = 63 for 24°C/control, and *n* = 61 for 24°C/exposed).

### 2.2 Data collection

To quantify host responses to the parasite at the earliest stages of infection, we examined animals exposed to parasites at the end of the 24‐h inoculation period under an Olympus BX53F compound microscope (200–400× magnification). We scanned the anterior and posterior of the gut, where spores are most likely found penetrating into the host's body cavity (Stewart Merrill et al., 2019). We counted the number of spores, split into two categories (sensu Stewart Merrill et al., 2019): embedded spores (i.e., partially embedded in the gut epithelium; Figure 1b) and hemocoel spores (i.e., penetrated into the body cavity; Figure 1b); this allows us to quantify gut resistance (i.e., the extent to which the gut epithelium acts as a barrier to infecting spores) as the number of embedded spores divided by the total number of attacking spores (embedded spores + hemocoel spores), as done in earlier studies (Stewart Merrill et al., 2019; Sun, Dziuba, Jaye, et al., 2022). Meanwhile, we determined gut epithelium thickness by averaging the height of three haphazardly selected epithelium cells at the anterior end of the gut (Sun, Dziuba, Jaye, et al., 2022). In *Daphnia* spp., the gut epithelium is one cell layer thick. In addition, to quantify the immune response, we counted the total number of hemocytes attaching to the hemocoel spores and determined the number of hemocytes per spore (total number of hemocytes divided by the number of hemocoel spores) (Stewart Merrill et al., 2019; Sun, Dziuba, Jaye, et al., 2022). At this point, we also determined the host body size by measuring the distance between the center of the eye and the base of the tail spine (cellSens Software; Olympus, version 1.18).

To determine host fitness, we checked all animals daily for mortality and counted the number of offspring produced, which were then removed from the beakers. All animals were fed three times a week (*A. falcatus* food, 20,000 cells/ml), and maintained in 50 ml filtered lake water that was changed once a week. Once the last infected individual was found dead, the unexposed animals were checked twice a week, since uninfected *Daphnia* live significantly longer than infected ones (Sun, Dziuba, Jaye, et al., 2022). We determined the lifespan of all animals upon their natural death. Dead infected animals were kept individually in a 1.5 ml tube of 100 μl deionized water and stored in a refrigerator before determining spore yield. We calculated two key components of parasite fitness: probability of terminal infections (that is, the proportion of infections that yielded transmission spores, which is the stage that is capable of infecting a new host) and spore yield per infected host (that is, the number of mature transmission spores per host). We determined the spore yield by grinding the host using a cordless pellet pestle (Fisherbrand; Fisher Scientific) for 60 seconds to release spores and homogenize the solution, then adding a 10 μl sample to a Neubauer hemocytometer. We averaged the number of mature spores from four grids for estimation of spore yield.

Animals that died within 7 days after exposure were excluded from the analysis because it was not possible to determine their infection status. Only the asexual phase of *D. dentifera* was considered in this study. Thus, we also excluded males, which occurred at a relatively low frequency (39 out of 287 total animals).

### 2.3 Data analysis

All analyses were performed in R (version 4.1.2; R Development Core Team, 2014) using generalized linear mixed models with the glmer function in the *lme4* package (version 1.1‐27.1; Bates et al., 2015). Analysis of variance was performed in the *car* package (version 3.0‐12; Fox et al., 2021). Additional packages used include the *coxme* package (version 2.2‐16; Therneau, 2012) for survival analyses, and the *emmeans* package (version 1.7.1‐1; Lenth, 2021) for Tukey post‐hoc comparisons once significant interaction terms were detected.

In most analyses, we included temperature (F0 Temperature) and parasite exposure (F0 Parasite) of the parental generation, and those of the offspring generation (F1 Temperature and F1 Parasite), as well as the interaction between the four variables (that is, F0 Temperature, F0 Parasite, F1 Temperature, F1 Parasite); exceptions to this are described below. In addition, parent ID was included as a random factor when analyzing data on offspring generation since the multiple offspring of the same clutch were used from the same mother.

We analyzed gut resistance (embedded spores divided by attacking spores, as described above) and hemocytes per spore [after ln(*x* + 1) transformation] with a Gaussian distribution. When analyzing gut resistance, we also included gut epithelium thickness as a covariate. These analyses of resistance to infection included all animals, including those that were exposed to spores but that did not develop terminal infections. For the remaining analyses, we only used unexposed (and, therefore, uninfected) animals and animals that were infected, excluding individuals that were exposed but uninfected. We analyzed age at first reproduction and first clutch size with a Poisson distribution, and lifetime fecundity with a negative binomial distribution to account for overdispersion. However, we note that we did not expect a within‐generation effect of parasite exposure on age at first reproduction or first clutch size, as the experimental animals likely deposited their first clutch in the brood chamber right around the time of parasite exposure; therefore, the results for age at first reproduction and first clutch size are presented in Appendix S1 (Figure S1). For the survival analysis, we analyzed host survival with a Cox proportional hazard mixed effect model. The assumptions of proportional hazard models were met by evaluating both graphically and using a goodness‐of‐fit test.

For the analysis of lifetime host reproduction, a further analysis was conducted since we were interested in the potential for a trade‐off between reproductive success and immune responses. Specifically, we were interested in whether a greater immune response (quantified as hemocytes per spore) would come at a cost of lifetime host reproduction. Thus, we additionally included hemocytes per spore as a covariate. We were also interested in whether this relationship would be impacted by within‐ or transgenerational impacts of elevated temperature or parasite exposure. Therefore, this analysis included gut resistance and hemocytes per spore as covariates, in addition to the fixed effects of the temperature of both parental and offspring generations (F0 and F1 Temperature) and parasite exposure of the parental generation (F0 Parasite); parasite exposure in the F1 generation was not included because all the individuals in this analysis were exposed to (and infected by) parasites in the F1 generation.

Finally, we were also interested in two key components of parasite fitness: the probability of terminal infection and spore yield per host. For terminal infection outcomes, we analyzed the probability of terminal infection (terminal infection: 1; no terminal infection: 0) with a binomial distribution and logit link function. Among animals that reached terminal infection, we analyzed the spore yield per host [ln(*x* + 1)] with a Gaussian distribution, and included gut resistance and hemocytes per spore as covariates.

## 3 RESULTS

### 3.1 Within‐ and transgenerational effects of stressors on host fecundity and survival

**FIGURE 3:**
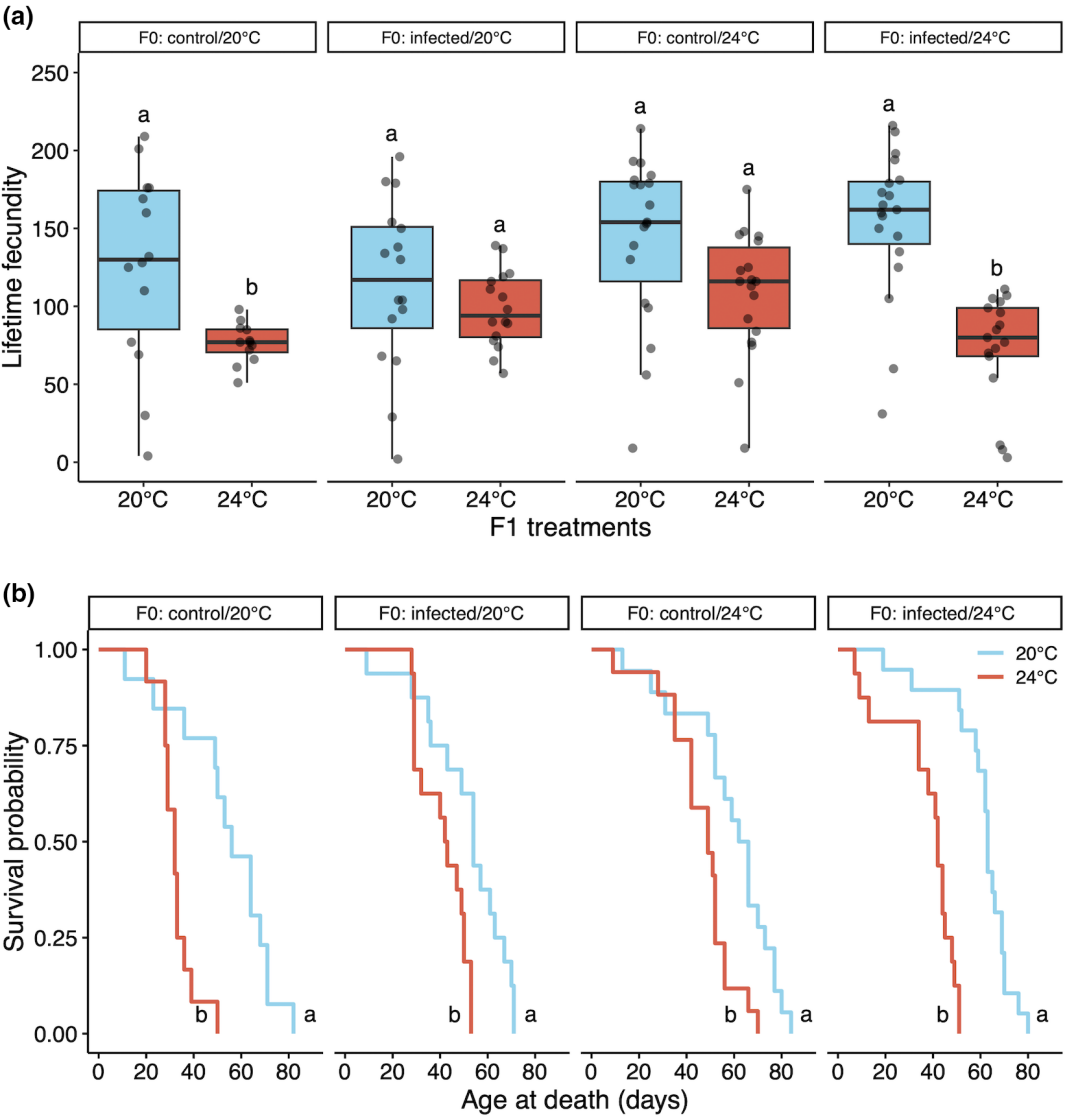
Within‐ and transgenerational effects of elevated temperature and parasite infection on host fecundity (a) and lifespan (b) of F1 individuals that were not exposed to parasites. Results for F1 individuals that were exposed to parasites can be found in Figure S2. The letters indicate statistically significant differences in pairwise comparisons between F1 treatments within the same F0 treatment. “F0” = parental generation, “F1” = offspring generation. The box plots in (a) show median values, the 25th and 75th percentiles, and interquartile ranges. Kaplan–Meier plots in (b) show host survival over a period of 84 days.

We detected within‐ and transgenerational effects of elevated temperature and parasite infection on lifetime fecundity, as evidenced by a significant interactive effect between parental and offspring environment for both elevated temperature and parasite infection (Table S1). The transgenerational impacts were most pronounced when offspring were not exposed to parasites (Figure 3a). If parents experienced neither stressor (left panel of Figure 3a) or both stressors (right panel of Figure 3a), offspring that were exposed to elevated temperature suffered lower fecundity as compared to those that were raised at ambient temperature (neither parental stressor: *z* = 2.78, *p* = .028; both parental stressors: *z* = 4.88, *p* < .001). By contrast, if the parents were only exposed to one stressor (either parasite exposure, as in the second panel of Figure 3a, or elevated temperature, as in the third panel of Figure 3a), offspring that were exposed to elevated temperature had the same fecundity as those raised at ambient temperature (parents exposed to parasites: *z* = 0.92, *p* = .795; parents exposed to elevated temperature: *z* = 1.84, *p* = .253). Overall, these results suggest that a single parental stressor helped offspring maintain high fecundity in the face of elevated temperature, but multiple parental stressors led to reduced offspring fitness at elevated temperature. The pattern for offspring exposed to parasites was much simpler: reproduction of infected offspring was consistently low across all parental environments (control/20°C: *z* = −2.11, *p* = .149; exposed/20°C: *z* = 0.61, *p* = .929; control/24°C: *z* = 1.49, *p* = .446; exposed/24°C: *z* = 2.19, *p* = .125; Figure S2a).

Lifespan was also influenced by both parental and offspring environments (Figure 3b; Figure S2b; Table S1). For offspring that were not exposed to parasites (shown in Figure 3b), elevated temperature shortened lifespan (red lines are to the left of blue lines in Figure 3b), but the extent of reduction was greater when their parents were reared under ambient temperature without parasite infection (*z* = −5.59, *p* < .001; left panel in Figure 3b) or when parents were exposed simultaneously to elevated temperature and parasite infection (*z* = −5.26, *p* < .001; right panel in Figure 3b). While elevated temperature also reduced the survival of unexposed individuals whose parents were exposed to elevated temperature but not parasites (*z* = −3.61, *p* = .002) or to parasite infection but not elevated temperature (*z* = −3.50, *p* = .003), this reduction was more modest (that is, the red lines on the two center panels in Figure 3b are not as far from the blue lines, as compared to the left and right panels). Furthermore, comparing the differences in lifespan of offspring exposed to elevated temperature alone, individuals whose parents were exposed singly to elevated temperature had higher survival probability compared to those exposed to both elevated temperature and parasite infection (*z* = −2.69, *p* = .036), and to those never exposed to any of these stressors before (*z* = 3.86, *p* < .001). Offspring infected by parasites (Figure S2b) died earlier than uninfected hosts (Figure 3b), with a greater lifespan reduction at elevated than ambient temperature when parents were exposed to stressful environments (exposed/20°C: *z* = −3.33, *p* = .005; control/24°C: *z* = −3.97, *p* < .001; exposed/24°C: *z* = −4.17, *p* < .001), although no difference was found when parents were unexposed to any stressor (*z* = 0.37, *p* = .983).

Overall, when offspring were not exposed to parasites (Figure 3), the offspring of mothers who were exposed to neither stressor or to both stressors suffered the most when exposed to elevated temperature, with reduced lifetime fecundity and shorter lifespans; by contrast, the elevated temperature had more modest impacts on the unexposed offspring of mothers who experienced only one of the two stressors. For offspring that were infected by the parasite (Figure S2), all individuals suffered strong reductions in fecundity and reductions in lifespan, as compared to uninfected individuals (Figure 3).

### 3.2 Within‐ and transgenerational effects on host immune responses

Gut resistance to attacking spores was similar across all parental and offspring treatments (Figure S3a; Table S1). By contrast, the number of hemocytes per spore was determined by the temperature in offspring generations (Figure S3b; Table S1). Specifically, elevated temperature consistently led to fewer hemocytes per spore in offspring generations.

### 3.3 Potential trade‐off between immune response and host reproduction

**FIGURE 4:**
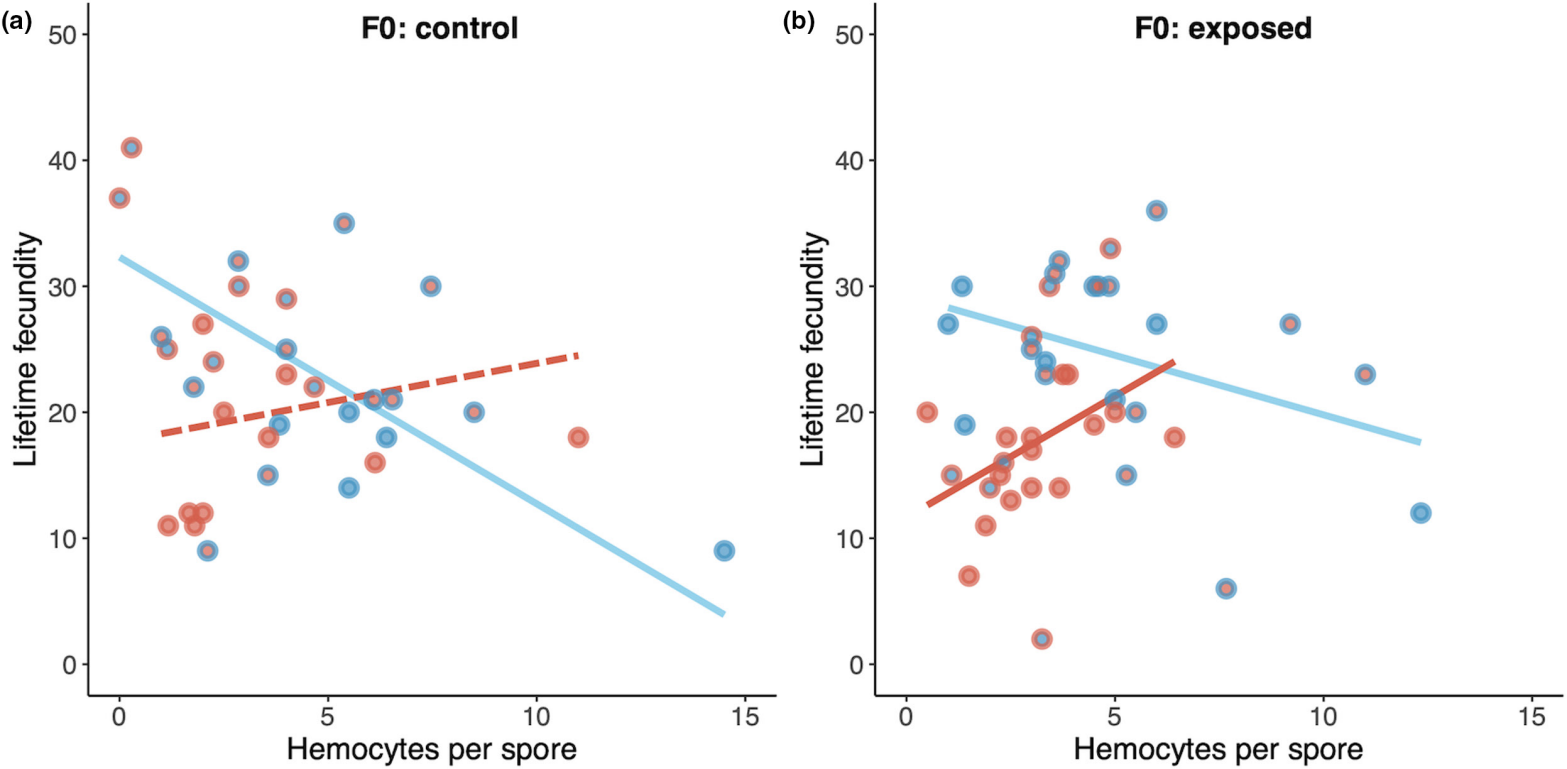
Within‐ and transgenerational effects of elevated temperature on the relationship between lifetime fecundity and hemocytes per spore in the offspring generation whose parental generations were unexposed (a) or exposed (b) to parasites. Solid and dashed lines represent significant and nonsignificant relationships predicted from GLMMs, respectively. Because both parental (F0) and offspring (F1) temperature influenced reproduction, fill colors denote temperature treatments of the parental generation (blue fills are for 20°C; red fills are for 24°C), and the outline colors denote temperature treatments of the offspring generation (blue outlines are for 20°C; red outlines are for 24°C). In both panels, the regression lines are grouped according to the results of the model; in (a), the regression lines are divided according to parental generation temperature (20°C F0 blue line, 24°C F0 red line), whereas in (b), the regression lines are divided according to offspring (F1) temperature.

Immune responses were correlated with lifetime fecundity but in opposite directions at ambient vs. elevated temperature (Figure 4; Table S3). Our statistical analysis suggests the hemocyte–lifetime fecundity relationship in the F1 generation is primarily associated with the parental temperature for the offspring of unexposed parents but with the offspring temperature if the parents were exposed to the parasite; as a result, we focus on these groupings. At ambient temperature, there is evidence of a trade‐off between investment in immune responses and reproduction: individuals that mobilized more hemocytes per spore had lower lifetime fecundity (Figure 4). Ambient temperature for unexposed parents resulted in offspring that had reduced fecundity with higher hemocyte production (*χ* ^2^ = 9.05, df = 1, *p* = .003; Figure 4a, blue line). At ambient temperature in the F1 generation for the offspring of exposed parents, there was again a negative relationship between hemocyte production and lifetime fecundity (*χ* ^2^ = 5.78, df = 1, *p* = .016; Figure 4b, blue line). By contrast, at elevated parental temperature, there was no significant relationship between immune response and fecundity for the offspring of parents who had not been exposed to parasites (unexposed: *χ* ^2^ = 0.27, df = 1, *p* = .602, Figure 4a red line); moreover, for the offspring of parents who had been exposed to parasites, individuals reared at an elevated temperature that mobilized more hemocytes per spore had higher lifetime fecundity (exposed: *χ* ^2^ = 1.99, df = 1, *p* = .047, Figure 4b red line).

### 3.4 Within‐ and transgenerational effects on terminal infection and spore yield

Temperature treatments did not influence the probability of terminal infection. Parental environment also did not influence the probability of terminal infection (Figure S4a; Table S2). For hosts that developed a terminal infection, the spore yield per host was lower at elevated temperatures (Figure S4b; Table S2); neither temperature nor parasite treatments during the parental generation had an effect.

## 4 DISCUSSION

Transgenerational plasticity can allow organisms to respond rapidly to changing environments, potentially protecting them from fitness loss associated with stressors (Donelson et al., 2018; Salinas & Munch, 2012; Uller, 2008). Yet, the ability of transgenerational plasticity to counteract the joint influence of biotic and abiotic stressors has been understudied, limiting our understanding of the role of transgenerational plasticity in a variable world. Here, we found that transgenerational plasticity induced by temperature and parasite stress influenced host performance. This effect was particularly prominent for offspring that were exposed to temperature stress but not parasitism: in this case, the offspring of mothers who were exposed to one stressor (either temperature or parasite stress) were better able to tolerate elevated temperature, as compared to the offspring of mothers who experienced neither or both stressors. However, parasite stress had much stronger negative effects on host fitness than temperature stress did, and the large reduction in host fitness arising from infection was not mitigated by transgenerational plasticity. Thus, transgenerational plasticity helped offspring maintain fitness in the face of elevated temperature if the parents had experienced only one stressor but did not protect offspring exposed to parasites. By contrast, parasite fitness was mostly unaffected by host transgenerational plasticity. Together, our results provide evidence of transgenerational plasticity, but the degree to which it benefitted the host depended on the identity and combination of environmental stressors.

Although thermal transgenerational plasticity has been subjected to rigorous experimental testing for its adaptive value in *Daphnia* spp., there is currently no consensus regarding whether it is always present, or about whether it tends to increase offspring fitness (Kielland et al., 2017; Walsh et al., 2014). While temperature stress tends to co‐occur with different types of stressors, such as parasite exposure, only a handful of studies have considered both (Garbutt et al., 2014; Hector et al., 2021), and, to our knowledge, none has thoroughly evaluated transgenerational interactions between parasite exposure and elevated temperature. Our results partially supported the environmental matching hypothesis (Paraskevopoulou et al., 2022), wherein parents prime their offspring to better deal with stressors. In our study, elevated temperature represented a stressful environment, reducing fecundity and lifespan. However, the offspring of parents who experienced elevated temperature suffered less (in terms of fecundity and lifespan) than did the offspring of parents who experienced ambient temperature. This finding differs from a finding on a different *Daphnia*‐parasite system (Hector et al., 2021), which found little effect on maternal temperature. Interestingly, the offspring of parents exposed to parasites also suffered less at elevated temperature compared with ambient temperature. One possible explanation for this is the potential for shared physiological responses, such as heat‐shock proteins; these maintain cellular stability and resistance to heat (Zhang et al., 2014), and, while named after their role in responding to heat stress, can be upregulated in response to a wide variety of stressors, including parasite exposure (Selbach et al., 2020). Alternatively, the elevated temperature may be an environmental signal of infection risk for natural *Daphnia* populations since both stressors are likely to co‐occur (Garbutt et al., 2014). Upregulated physiological responses to heat stress in response to parasite infection are common in many taxa, including fish, birds, and mammals (Forsyth et al., 1997; Martinez et al., 1999; Merino et al., 1998). However, the offspring of parents who were simultaneously exposed to temperature and parasite stressors suffered the full negative impacts of elevated temperature. Together, these results suggest that transgenerational effects can help organisms cope with changing environmental conditions. Yet, our results also suggest there may be a limit to the ability of transgenerational plasticity to protect offspring in more stressful environments, possibly because resources, which must be allocated simultaneously to both stressors, are limited (Bubliy et al., 2012).

Beyond the finding that all infected hosts suffered large reductions in fecundity and lifespan (Figure S2), as expected given the known virulence of this parasite, two other patterns related to infection stand out. First, elevated temperature led to a lower immune response, on average, with fewer hemocytes recruited per penetrated spore (Figure S3b). Second, the nature of the relationship between immune responses and host fecundity reversed under elevated temperature (Figure 4). We hypothesized that there might be a trade‐off between fecundity and immune responses, as it has been seen in many other systems (Gwynn et al., 2005; Schwenke et al., 2016); such a trade‐off could arise if mounting a strong immune response prevents hosts from investing as many resources in reproduction. At ambient temperature, a stronger immune response was indeed associated with lower reproductive success, irrespective of parental exposure to parasites (Figure 4). Surprisingly, this trade‐off disappeared under elevated temperature: the fecundity–immune response relationship was flattened when the parental generation experienced elevated temperature but was not exposed to parasites (Figure 4a) and became positive when offspring encountered elevated temperature and when parents had been exposed to parasites (Figure 4b). This suggests that parents who were exposed to parasites can potentially prime offspring generation to face the joint stressors of both elevated temperature and parasite infection. The exact mechanism of such immune priming effect has yet to be investigated but might occur via epigenetic inheritance altering offspring gene expression (Curley et al., 2011), or transfer of immune components and pathogen‐associated molecular patterns (Roth et al., 2018). These findings highlight the importance of considering transgenerational effects in response to different environmental challenges when exploring trade‐offs, and the importance of incorporating multiple fitness components to evaluate the adaptive value of transgenerational effects.

Although physical and immune responses are two potent defenses against parasite infection, we instead found that neither gut resistance nor hemocytes per spore explain differences in spore yield per host. Elevated temperature also had negligible effects on the probability of infection and spore production for hosts that were infected, except that infected hosts generally produced fewer spores when the offspring generation was exposed to elevated temperature. These findings, alongside the effects of temperature on hosts, suggest that elevated temperature and parasites mainly acted independently in affecting the host's fitness components, but temperature can indirectly alter the direction of the fecundity–immune response relationship via within‐ and transgenerational effects.

Our results show that transgenerational plasticity helped individuals cope with elevated temperature. However, this only occurred when parents were singly stressed (by either the temperature or parasite stressor). The offspring of parents simultaneously exposed to both stressors suffered large fitness reductions when exposed to elevated temperature, potentially revealing a limit of adaptive transgenerational plasticity. Moreover, the identity of the stressor clearly matters: transgenerational plasticity did not protect individuals from the virulent effects of the parasite. Furthermore, our results demonstrate the importance of considering multiple fitness‐associated traits to understand the adaptive values of transgenerational plasticity induced by multiple stressors in a changing world: adaptive transgenerational plasticity might be masked without a complete screening of key traits involving performance trade‐offs. Future studies identifying the molecular mechanisms, e.g., epigenetic modifications, at various stages of ontogeny (Donelan et al., 2020) would be particularly valuable in order to help improve our understanding of the role of transgenerational plasticity in a rapidly changing world.

## AUTHOR CONTRIBUTIONS

**Syuan‐Jyun Sun:** Conceptualization (lead); data curation (lead); formal analysis (lead); investigation (lead); methodology (lead); project administration (lead); resources (lead); validation (lead); visualization (lead); writing – original draft (lead); writing – review and editing (lead). **Marcin K. Dziuba:** Conceptualization (lead); data curation (lead); formal analysis (supporting); investigation (lead); methodology (lead); project administration (lead); resources (supporting); validation (supporting); visualization (supporting); writing – original draft (supporting); writing – review and editing (supporting). **Riley N. Jaye:** Conceptualization (supporting); data curation (supporting); investigation (supporting); methodology (equal); validation (supporting); visualization (supporting); writing – original draft (supporting); writing – review and editing (supporting). **Meghan A. Duffy:** Conceptualization (lead); data curation (supporting); formal analysis (supporting); funding acquisition (lead); investigation (lead); methodology (supporting); project administration (lead); resources (lead); supervision (lead); validation (lead); visualization (supporting); writing – original draft (lead); writing – review and editing (lead).

## CONFLICT OF INTEREST

The authors declare no competing interests.

## Supporting information


Appendix S1.

## Data Availability

The dataset and R scripts are openly available on GitHub (https://github.com/syuanjyunsun/host_trans_plasticity) and Dryad (https://doi.org/10.5061/dryad.4qrfj6qf5).
