# Supplementary material for: Transgenerational plasticity in a zooplankton in response to elevated temperature and parasitism
Source: Ecol Evol. 2023 Feb 3;13(2):e9767. doi: 10.1002/ece3.9767 (PMC9897957; doi:10.1002/ece3.9767)
Supplement: Supplementary file 1 — Appendix S1. [file ECE3-13-e9767-s001.docx]

**Appendix 1**

**Transgenerational plasticity in a zooplankton in response to elevated temperature and parasitism**

Syuan-Jyun Sun^1,2,*^, Marcin K. Dziuba^1^, Riley N. Jaye^1^, and Meghan A. Duffy^1^

^1^Department of Ecology & Evolutionary Biology, University of Michigan, Ann Arbor, MI 48109, USA

^2^International Degree Program in Climate Change and Sustainable Development, National Taiwan University, Taipei 10617, Taiwan

*Supplementary results related to host reproduction*

Age at first reproduction

We observed within- and trans-generational impacts of elevated temperatures on age at first reproduction. Both parental and offspring temperature impacted age at first reproduction (Fig. S2A; Table S1). The earliest reproduction was by individuals raised at 24°C whose mothers had also been raised at 24°C, while the latest first reproduction was by individuals raised at 20°C whose mothers had also been raised at 20°C. In contrast to the temperature results, parasite exposure in the parental or offspring generation did not significantly impact age at first reproduction (Fig. S2A; Table S1).

First clutch size

Neither temperature elevation nor parasite infection in the parental or offspring generation significantly impacted first clutch size. Instead, first clutch size was similar across all parental and offspring treatments (Fig. S2B; Table S1).


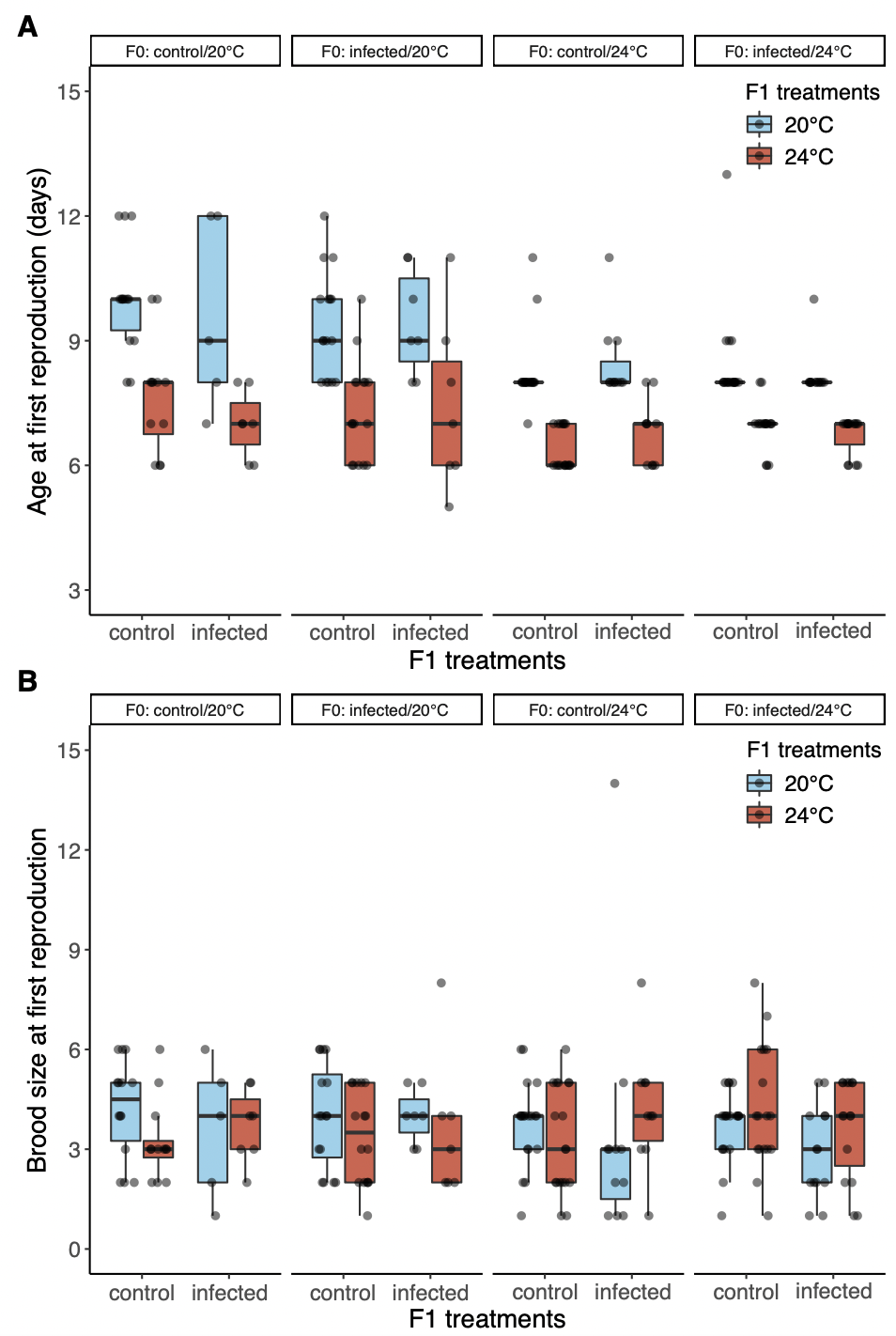


Figure S1. Within- and trans-generational effects of temperature elevation and parasite infection on host age at first reproduction (**A**) and brood size at first reproduction (**B**). “F0” = parental generation, “F1” = offspring generation. The box plots show median values, the 25^th^ and 75^th^ percentiles, and interquartile ranges.


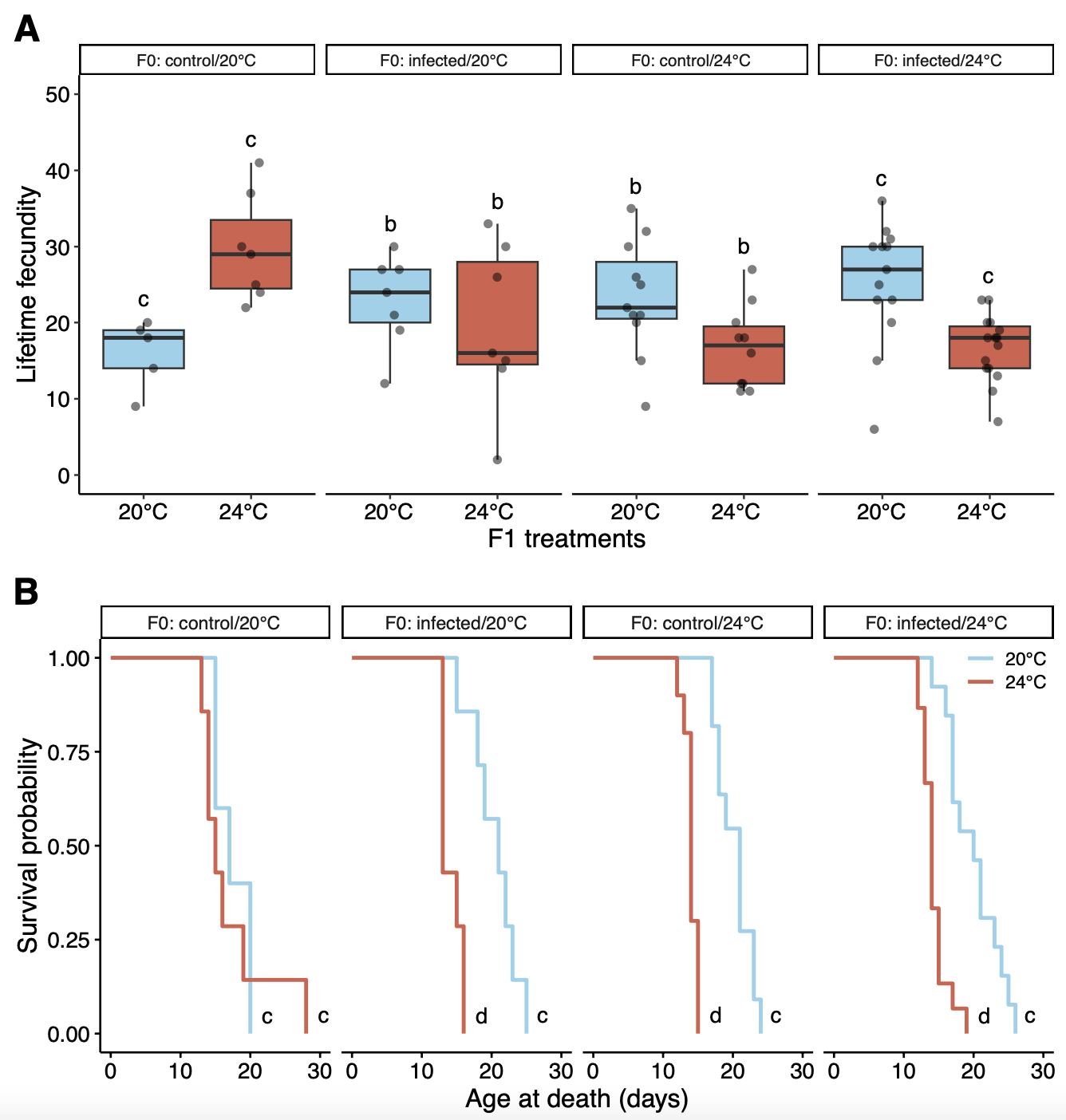


Figure S2. Within- and trans-generational effects of elevated temperature and parasite infection on host fecundity (**A**) and lifespan (**B**) of F1 individuals that were exposed to parasites. The letters indicate statistically significant differences of pairwise comparisons between F1 treatments within the same F0 treatment. “F0” = parental generation, “F1” = offspring generation. The box plots in (**A**) show median values, the 25^th^ and 75^th^ percentiles, and interquartile ranges. Kaplan-Meier plots in (**B**) show host survival over a period of 28 days.


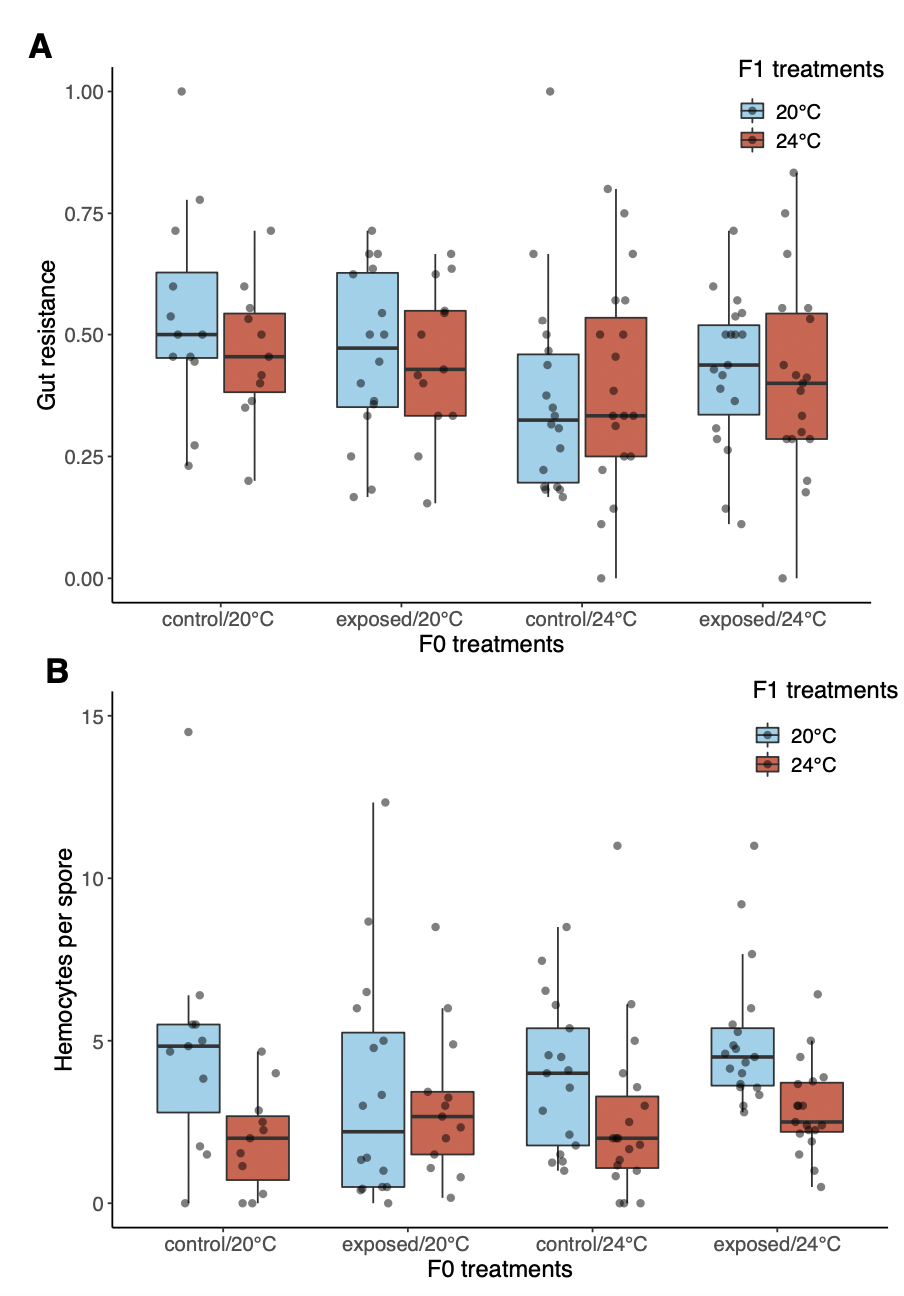


Figure S3. Within- and trans-generational effects of temperature elevation and parasite infection on host defense measured as gut resistance (**A**) and number of hemocytes per spore (**B**). “F0” = parental generation, “F1” = offspring generation. The box plots show median values, the 25^th^ and 75^th^ percentiles, and interquartile ranges.

Figure S4. Within- and trans-generational effects of elevated temperature and parasite infection on the probability of terminal infection (**A**) and spore yield per host (ln (spores+1)) (**B**). Means and standard error bars are shown in (**A**). The box plots in (**B**) show median values, the 25^th^ and 75^th^ percentiles, and interquartile ranges. “F0” = parental generation, “F1” = offspring generation.

| Table S1. Host defense and life history traits in the offspring generation (F1) explained by the effects of temperature (F0 Temp) and parasite exposure (F0 Para) in the parental generation (F0), and by the effects of temperature (F1 Temp) and parasite exposure (F1 Para) in the offspring generation. Statistically significant p values are highlighted in bold. | | | | |
| --- | --- | --- | --- | --- |
| **Dependent variable** | **Explanatory variables** | ***X^2^*** | **d.f.** | ***p* value** |
| Gut resistance | F0 Temp | 3.69 | 1 | 0.055 |
|  | F1 Temp | 0.19 | 1 | 0.662 |
|  | F0 Para | 0.00 | 1 | 0.966 |
|  | Gut thickness | 0.01 | 1 | 0.906 |
| Hemocytes per spore | F0 Temp | 2.78 | 1 | 0.096 |
|  | F1 Temp | 13.25 | 1 | **<0.001** |
|  | F0 Para | 1.30 | 1 | 0.254 |
| Age at first reproduction | F0 Temp | 6.14 | 1 | **0.013** |
|  | F1 Temp | 22.40 | 1 | **<0.001** |
|  | F0 Para | 0.003 | 1 | 0.960 |
|  | F1 Para | 0.02 | 1 | 0.890 |
| Brood size at first reproduction | F0 Temp | 0.03 | 1 | 0.855 |
|  | F1 Temp | 0.09 | 1 | 0.767 |
|  | F0 Para | 0.01 | 1 | 0.918 |
|  | F1 Para | 0.23 | 1 | 0.633 |
| Lifespan | F0 Temp | 0.78 | 1 | 0.377 |
|  | F1 Temp | 31.21 | 1 | **<0.001** |
|  | F0 Para | 0.92 | 1 | 0.337 |
|  | F1 Para | 61.12 | 1 | **<0.001** |
|  | F0 Temp x F1 Temp | 5.16 | 1 | **0.023** |
|  | F0 Temp x F0 Para | 0.04 | 1 | 0.843 |
|  | F1 Temp x F0 Para | 5.08 | 1 | **0.024** |
|  | F0 Temp x F1 Para | 0.21 | 1 | 0.644 |
|  | F1 Temp x F1 Para | 13.51 | 1 | **<0.001** |
|  | F0 Para x F1 Para | 2.35 | 1 | 0.125 |
|  | F0 Temp x F1 Temp x F0 Para | 6.88 | 1 | **0.009** |
|  | F0 Temp x F1 Temp x F1 Para | 12.63 | 1 | **<0.001** |
|  | F0 Temp x F0 Para x F1 Para | 0.81 | 1 | 0.370 |
|  | F1 Temp x F0 Para x F1 Para | 11.10 | 1 | **0.001** |
|  | F0 Temp x F1 Temp x F0 Para x F1 Para | 11.36 | 1 | **0.001** |
| Fecundity | F0 Temp | 0.66 | 1 | 0.417 |
|  | F1 Temp | 7.70 | 1 | **0.006** |
|  | F0 Para | 0.37 | 1 | 0.542 |
|  | F1 Para | 63.29 | 1 | **<0.001** |
|  | F0 Temp x F1 Temp | 0.91 | 1 | 0.339 |
|  | F0 Temp x F0 Para | 0.58 | 1 | 0.448 |
|  | F1 Temp x F0 Para | 2.11 | 1 | 0.146 |
|  | F0 Temp x F1 Para | 0.60 | 1 | 0.440 |
|  | F1 Temp x F1 Para | 10.59 | 1 | **0.001** |
|  | F0 Para x F1 Para | 1.82 | 1 | 0.177 |
|  | F0 Temp x F1 Temp x F0 Para | 6.46 | 1 | **0.011** |
|  | F0 Temp x F1 Temp x F1 Para | 7.23 | 1 | **0.007** |
|  | F0 Temp x F0 Para x F1 Para | 1.12 | 1 | 0.291 |
|  | F1 Temp x F0 Para x F1 Para | 5.97 | 1 | **0.015** |
|  | F0 Temp x F1 Temp x F0 Para x F1 Para | 6.65 | 1 | **0.010** |

| Table S2. Effects of temperature on the parasite fitness components of infection outcomes in the offspring generation (F1). Statistically significant p values are highlighted in bold. | | | | |
| --- | --- | --- | --- | --- |
| **Dependent variable** | **Explanatory variables** | ***X^2^*** | **d.f.** | ***p* value** |
| Probability of terminal infection | F0 Temperature | 1.23 | 1 | 0.268 |
|  | F1 Temperature | 0.01 | 1 | 0.907 |
|  | F0 Parasite | 1.24 | 1 | 0.266 |
| Spore yield per host | F0 Temperature | 0.02 | 1 | 0.897 |
|  | F1 Temperature | 4.30 | 1 | **0.038** |
|  | F0 Parasite | 0.12 | 1 | 0.730 |
|  | Gut resistance | 3.78 | 1 | 0.052 |
|  | Hemocytes per spore | 0.01 | 1 | 0.936 |

| Table S3. Host lifetime fecundity and its relationship with immune responses in the offspring generation (F1) explained by the effects of temperature (F0 Temp) and parasite exposure (F0 Para) in the parental generation (F0), and by the effect of temperature (F1 Temp) in the offspring generation. Statistically significant p values are highlighted in bold. | | | | |
| --- | --- | --- | --- | --- |
| **Dependent variable** | **Explanatory variables** | ***X^2^*** | **d.f.** | ***p* value** |
| Fecundity | F0 Temp | 3.03 | 1 | 0.082 |
|  | F1 Temp | 1.80 | 1 | 0.179 |
|  | F0 Para | 0.002 | 1 | 0.966 |
|  | Gut resistance | 1.48 | 1 | 0.225 |
|  | Hemocytes per spore | 5.83 | 1 | **0.016** |
|  | F1 Temp x F0 Temp | 11.06 | 1 | **0.001** |
|  | F0 Temp x F0 Para | 6.98 | 1 | **0.008** |
|  | F1 Temp x F0 Para | 13.30 | 1 | **<0.001** |
|  | F0 Temp x Hemocytes per spore | 9.53 | 1 | **0.002** |
|  | F1 Temp x Hemocytes per spore | 0.27 | 1 | 0.605 |
|  | F0 Para x Hemocytes per spore | 0.29 | 1 | 0.590 |
|  | F0 Temp x F0 Para x Hemocytes per spore | 6.01 | 1 | **0.014** |
|  | F1 Temp x F0 Para x Hemocytes per spore | 10.21 | 1 | **0.001** |
